# Evaluation of the Ability to Predict Subsequent Metastasis of Early Oral Squamous Cell Carcinoma Using PET Radiomics Machine Learning Models

**DOI:** 10.3390/cancers17213573

**Published:** 2025-11-05

**Authors:** Yutaka Nikkuni, Hideyoshi Nishiyama, Masaki Takamura, Taichi Kobayashi, Marie Soga, Makiko Ike, Kouji Katsura, Takafumi Hayashi

**Affiliations:** Division of Oral and Maxillofacial Radiology, Graduate School of Medical and Dental Sciences, Niigata University, Niigata 951-8510, Japan; nisiyama@dent.niigata-u.ac.jp (H.N.); takamura@dent.niigata-u.ac.jp (M.T.); taichi@dent.niigata-u.ac.jp (T.K.); m-soga@dent.niigata-u.ac.jp (M.S.); m-ike@dent.niigata-u.ac.jp (M.I.); katsu@dent.niigata-u.ac.jp (K.K.); hayashi@dent.niigata-u.ac.jp (T.H.)

**Keywords:** radiomics, machine learning model, ^18^F-FDG-PET, early-stage oral squamous cell carcinoma, subsequent metastasis

## Abstract

**Background/Objectives**: Oral squamous cell carcinoma (OSCC) carries a risk of late metastasis not only in advanced stages but also in early stages. In this study, we built and tested radiomics-based machine learning (ML) models for predicting the risk of metastasis from early OSCC on ^18^F-FDG positron emission tomography (PET). **Methods**: Patients diagnosed with T1 or T2 squamous cell carcinoma who underwent a preoperative ^18^F-FDG PET-CT examination at a single institution between 2016 and December 2022 were included in this retrospective study. The presence or absence of late cervical lymph node metastasis was confirmed for all patients. Among the radiomics features extracted from the images, we selected those that were useful for predicting late metastasis and used them to create ML models. We then verified the prediction accuracy of the models. **Results**: A total of 109 subjects were included, of which 31 had late lymph node metastasis and 78 were without metastasis. The most accurate ML model created using radiomics features selected from the subject cases had an area under the curve of 0.977 and accuracy of 87.5%. **Conclusions**: We confirmed that ML models using radiomics features extracted from PET images can be useful for predicting late metastasis in patients with early-stage OSCC.

## Simple Summary

The prognosis following treatment for oral squamous cell carcinoma (OSCC) is affected by the presence or absence of subsequent lymph node metastasis, and this includes early-stage cancer. In this study, we created and tested radiomics-based machine learning (ML) models for predicting the likelihood of late metastasis from early-stage OSCC using ^18^F-FDG positron emission tomography (PET). A total of 109 patients who had been histopathologically diagnosed with early-stage OSCC and underwent PET examinations were the subjects of this study. We investigated whether radiomics features extracted from the tumor areas shown on PET images could predict the presence or absence of subsequent metastasis within one year. The results confirmed that a machine learning prediction model using radiomics features is useful for predicting subsequent metastasis of early-stage oral squamous cell carcinoma, and it was shown that it could contribute to determining treatment plans.

## 1. Introduction

### 1.1. Background

Although oral cancer is a relatively rare cancer, accounting for only 1% to 2% of all cancers, its risk cannot be ignored, because it is still associated with many recorded cases and deaths worldwide [1]. Oral cancer, especially oral squamous cell carcinoma (OSCC), carries a risk of subsequent lymph node metastasis even after treatment, which reduces the prognosis following treatment. Follow-up that includes imaging is required to detect subsequent cervical lymph node metastasis after OSCC treatment [2]. Early-stage OSCC is also at risk of developing cervical lymph node metastasis, as is advanced OSCC. However, there are conflicting opinions regarding the optimal treatment strategy for early-stage OSCC, especially N0 cases. Selective neck dissection has been suggested as a treatment strategy, but it may cause unnecessary invasiveness to the patient [3]. If the risk of subsequent metastasis from early-stage OSCC could be accurately predicted using pre-treatment imaging information alone, this invasive procedure could frequently be avoided, and useful information for determining treatment strategies would be provided. However, with current diagnostic imaging techniques, it is difficult to predict post-treatment metastases prior to treatment. Pretreatment positron emission tomography (PET) examinations provide highly accurate information about the primary tumor and cervical lymph node metastasis in patients with OSCC [4]. With the rapid increase in medical image information in recent years, a new analysis method called radiomics is attracting attention from many researchers [5,6]. It is a high-throughput analysis method that involves extracting many features from medical images and is useful for the phenotyping of lesions and improving the accuracy of diagnosis, differentiation, and prediction. The quantitative parameters obtained through radiomics analysis, which are referred to as radiomics features, include those that quantify minute texture features in an image. The typical focus of current radiomics research is to create machine learning (ML) predictive models from the vast quantity of measured radiomics features to improve the accuracy of conventional image-based diagnosis. ML models typically involve creating an algorithm that combines multiple quantitative parameters related to the target to provide prediction or discrimination. Such methods have also been introduced in the field of medical statistics where they have contributed to improving prediction accuracy [7,8,9,10].

### 1.2. Literature Survey

Many studies have used radiomics-based ML models to evaluate the histological type of OSCC and the presence of lymph node metastasis [11,12,13,14]. It is also reported that PET is useful for evaluating early-stage OSCC [15,16,17]. Several studies have reported that PET-based radiomics ML models can improve the diagnostic accuracy for OSCC on PET [18,19].

### 1.3. The Purpose of This Study

Based on the above findings, we created ML models using the features obtained from radiomics analysis of PET images and evaluated their accuracy for predicting subsequent cervical lymph node metastasis after surgery for early OSCC. If this study confirms that predictions using radiomics-based machine learning models can achieve sufficient accuracy, such models may provide valuable additional information prior to treatment for patients with early-stage oral squamous cell carcinoma, who are at low risk of subsequent metastasis and therefore susceptible to missed diagnoses. This predictive insight could help mitigate potential risks associated with undetected metastasis.

In studies involving large volumes of medical imaging data to predict post-treatment lesion prognosis, deep learning has been reported to be highly effective in numerous investigations [20,21]. However, predictive modeling using deep learning faces the challenge of interpretability—often referred to as the “black box” problem—wherein the behavior of AI systems remains difficult for humans to understand and explain. To address this issue, research into explainable AI (XAI) has been actively pursued [22]. Nevertheless, attempts to elucidate the imaging features of lesions based on their histopathological characteristics remain limited. By contrast, when constructing machine learning prediction models using radiomic features without deep learning, it may be possible to interpret the relationship between the selected radiomic features and histopathological findings [12,23]. Therefore, in the present study, we developed a machine learning prediction model using only radiomic features, without employing deep learning, and evaluated its predictive performance.

### 1.4. Sections of This Article

This paper is composed of the following sections:

Section 1: Background and purpose of this study;

Section 2: Subjects and methods of this study;

Section 3: Results of this study;

Section 4: Discussion of the results and limitations of this study;

Section 5: Conclusions of this study and future work.

## 2. Materials and Methods

### 2.1. Ethical Approval

This was a retrospective epidemiological study involving humans and was approved by the Ethics Committee of BLINDED (approval number 2022-0329). It adhered to the principles set out in the Declaration of Helsinki and ensured the protection of the subjects’ rights.

### 2.2. Subjects

**Figure 1:**
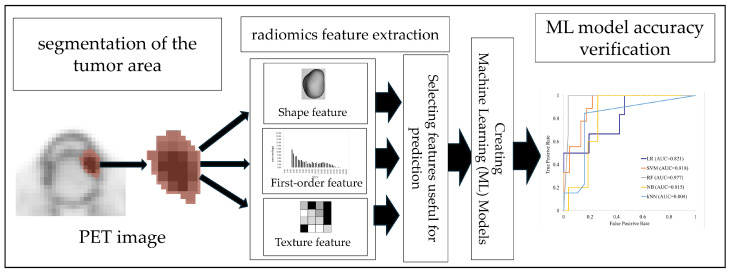
The flow diagram defining the steps of the tasks in this study.

The flow diagram defining the steps of the tasks in this study is shown in Figure 1. The subject inclusion criteria were as follows: (1) clinical diagnosis of oral cancer at BLINDED between July 2016 and July 2023; (2) subjects underwent a preoperative ^18^F-FDG PET/computed tomography (CT) examination; (3) the primary lesion was histopathologically diagnosed as squamous cell carcinoma of size T1 or T2 according to the TNM classification after surgery; and (4) post-operative follow-up confirming the presence or absence of subsequent metastasis to cervical lymph nodes. Cases in which lesions could not be identified on PET images or in which features could not be extracted by the radiomics feature extraction process were excluded. The minimum follow-up period for detecting subsequent metastasis was set at within one year postoperatively. The diagnostic criterion for lymph node metastasis used to evaluate subsequent metastasis was defined as the presence of at least one metastatic lymph node identified by histopathological examination of lymph nodes following neck dissection.

### 2.3. Image Acquisition

^18^F-FDG PET/CT imaging for preoperative diagnosis was carried out using a biograph mCT Flow 20 scanner (SIEMENS Healthineers, Erlangen, Germany). The spatial resolution of the images was 4.07 mm in the anteroposterior direction, 4.07 mm in the lateral direction, and 2.00 mm in the vertical direction, and the imaging range was from the skull base to the middle of the thigh.

### 2.4. Radiomics Analysis

Radiomics analysis was performed using the following steps: (1) segmentation of the tumor area, (2) extraction of radiomics features from the segmented tumor area, (3) identification of those radiomics features useful for predicting subsequent lymph node metastasis, (4) radiomics feature selection for ML model creation, and (5) evaluation of the accuracy of predictions by the ML models for subsequent metastasis of early OSCC. The open-source free software platform 3D slicer, version 5.3.1, was used for step (1); the open-source Python package PyRadiomics, version 3.0.1, was used for step (2); and the free software orange was used for steps (3) to (5).

As a first step, images were transformed to a 1 mm isotropic voxel resolution to avoid bias or distortion in the radiomics due to the original non-isotropic dimensions affecting specific cross-sections. Then, segmentation was performed to define a region of interest representing the tumor area from which to obtain the radiomics. This segmentation process first applied a standardized uptake value (SUV) threshold of 2.5 or higher to help define lesions on the PET images, as adopted in many previous studies [24]. The area of the primary tumor on the PET images was segmented twice on separate days by an oral and maxillofacial radiologist (Y.N.) with 19 years of experience, based on clinical information within the area with an SUV threshold of 2.5 or higher.

**Figure 2:**
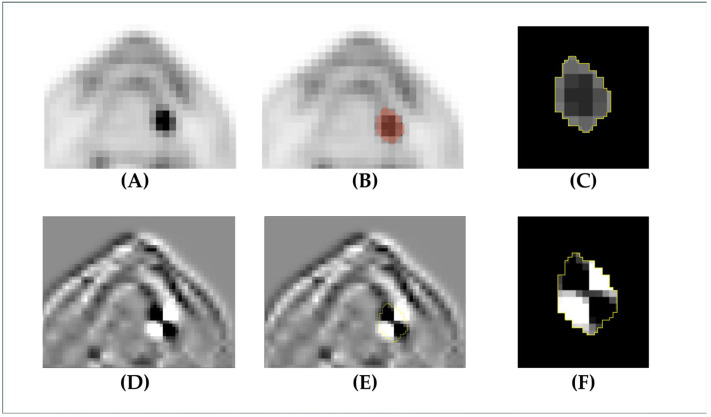
A case of early-stage (T1N0) cancer of the left tongue. In the PET image, regions with high FDG uptake are represented in black, and the lesion is identified as a black area (**A**). Based on image (**A**), tumor segmentation was performed using an SUV threshold of 2.5, resulting in image (**B**). The final segmented tumor region is shown in image (**C**). Image (**D**) is a wavelet-transformed version of image (**A**), which is used for radiomic feature extraction. Image (**E**) shows the tumor region segmented on the wavelet-transformed image (**D**), and the final segmented region is presented in image (**F**).

Radiomics features were obtained from the segmented region using the open-source Python package PyRadiomics, version 3.0.1. The extracted radiomics features included shape features, first-order features, and texture features, as used in many previous radiomics studies. Shape features quantify three-dimensional regions of interest on PET images using various scales, and indicate the three-dimensional metabolic features of the primary tumor extracted using a threshold of 2.5. First-order features included the mean, maximum, standard deviation, kurtosis, and skewness obtained by histogramming the distribution of the SUVs within a region. Texture features were obtained by converting unique texture patterns shown in an image composed of gray levels into matrices and measuring them so that they can be quantitatively evaluated. For the radiomics analysis in this study, five matrices calculated from the images were used to extract texture features: Gray Level Co-occurrence Matrix (GLCM), Gray Level Dependence Matrix (GLDM), Gray Level Run Length Matrix (GLRLM), Gray Level Size Zone Matrix (GLSZM), and Neighboring Gray Tone Difference Matrix (NGTDM). First-order features and texture features were also extracted from wavelet-transformed images whose XYZ components were composed only of high-frequency (named WL_HHH) or low-frequency components (named WL_LLL). The process of image extraction and processing, from region-of-interest segmentation to wavelet transformation, is presented in Figure 2. The metabolic distribution of early OSCC that causes subsequent metastasis may have a texture pattern with either large or small changes in SUV distribution on PET images. Therefore, texture features were obtained using 13 bins as follows: 0.01, 0.02, 0.03, 0.05, 0.1, 0.2, 0.3, 0.5, 1, 2, 3, 5, and 10. Finally, we obtained 2993 radiomics features: 14 morphological features, 54 first-order features, and 2925 texture features. The radiomics features obtained varied greatly in scale, and because it was necessary to exclude this scale difference for ML model creation, we z-score-normalized all features to a mean of 0 and standard deviation of 1.

**Figure 3:**
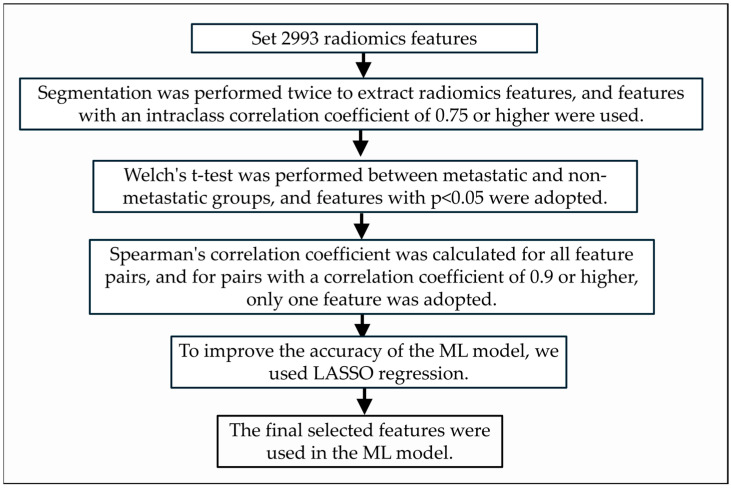
Flow chart for radiomics feature selection for ML model creation.

To evaluate the reproducibility of the feature values obtained from the segmented regions, we calculated the intraclass correlation coefficient (ICC) between the values obtained in the first and second segmentation. Only features with a value of 0.75 or higher, indicating a high correlation, were adopted. To assess the predictive utility of individual radiomics features for subsequent metastasis in early-stage oral squamous cell carcinoma, patients were divided into two groups: those who developed subsequent metastasis (metastasis group) and those who did not (non-metastasis group). Statistical significance between the two groups was evaluated for each radiomics feature. Assuming that the distribution of each feature was non-normal, Welch’s t-test was employed for the analysis. A *p*-value of less than 0.05 was considered statistically significant, indicating that the feature was potentially useful for predicting subsequent metastasis. Features showing significant differences were considered useful for predicting subsequent metastasis and were used to create the ML models. If multiple mutually correlated features are selected for ML model creation, the performance of the ML model may decrease; i.e., the features used in ML model creation need to be independent features with no redundancy. Therefore, the Spearman correlation coefficient was calculated between all pairs of features that showed a significant difference in the Welch’s t-test, and if a pair showed a value of 0.9 or higher, only one of the pairs was adopted. We used the least absolute shrinkage and selection operator (LASSO) regression model to eliminate excess features that could cause overfitting in the ML model, after which the final selected optimal features were used for ML model creation. Figure 3 shows the process of extracting radiomics features from the segmented regions and selecting them to build a machine learning model. The ML models used in this evaluation were logistic regression, support vector machine, random forest, naive Bayes, and k-nearest neighbor algorithms. LR is a regression analysis model that takes multiple features as input and calculates a function that outputs the probability of a true prediction of one of two or more discrete classes. SVM is a machine learning model that uses margin maximization and the kernel method to create a “classifier” that determines a boundary line or hyperplane that divides two classes of data based on training data. This model calculates separation using a hyperplane that maximizes the distance (margin) from the sample closest to the boundary. A kernel function is an algorithm used to increase dimensionality and separation by adding nonlinear features to data representation. RF is a machine learning model that creates many decision trees and predicts the classification of unknown data based on the majority vote of these trees. NB is a machine learning model that calculates the probabilities of all estimates given data and outputs the one with the highest probability as the estimation result. It is assumed that the data features are independent and uncorrelated with each other, and each feature independently affects the estimation results. KNN is a method of dividing data into groups by inferring which group the target data belongs to using a majority vote of the surrounding data.

For the creation and validation of the ML model, the total number of cases were randomly divided 70%/30% into the training dataset to create the ML model and the test dataset to verify its performance, respectively. To evaluate the performance of each ML model, we calculated the receiver operating characteristics curve (ROC), area under the curve (AUC), accuracy, sensitivity, specificity, positive predictive value (PPV), and negative predictive value (NPV) of each model for predicting subsequent metastasis.

## 3. Results

**Figure 4:**
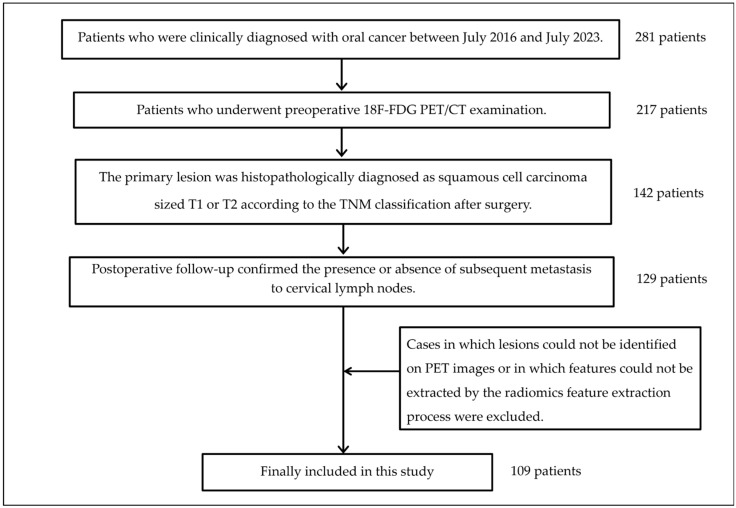
Flow chart for patient inclusion and exclusion.

**Figure 5:**
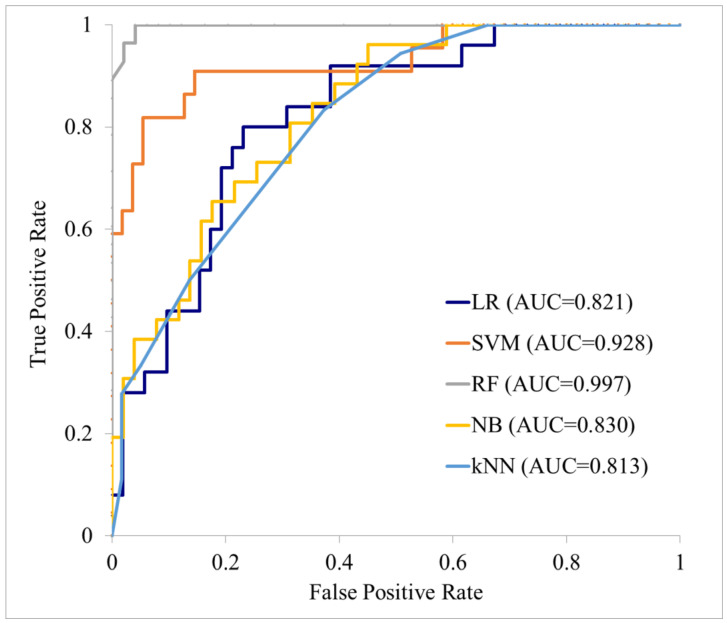
Performance of each machine learning model on the training data. LR: logistic regression; SVM: support vector machine; RF: random forest; NB: naive Bayes; KNN: k-nearest neighbor.

**Figure 6:**
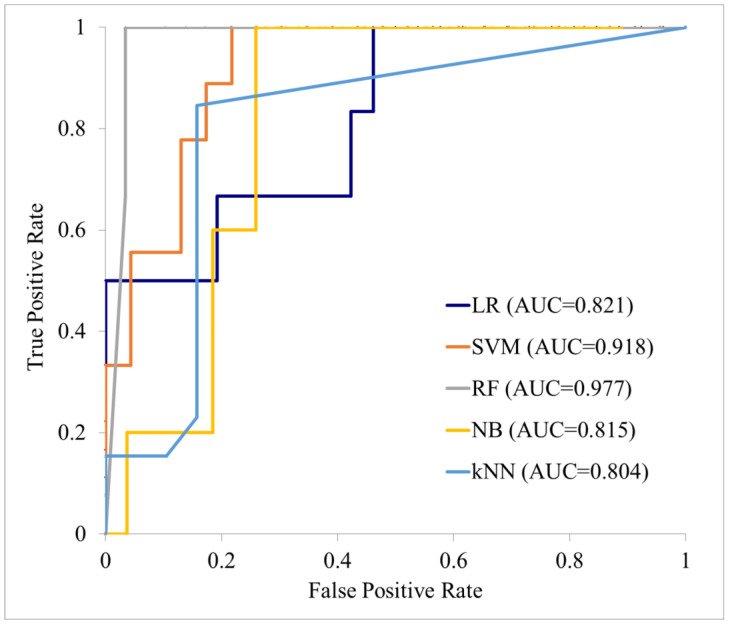
Performance of each machine learning model on the test data. LR: logistic regression; SVM: support vector machine; RF: random forest; NB: naive Bayes; KNN: k-nearest neighbor.

In this study, 109 patients met the inclusion criteria, of which 31 and 78 were assigned to the subsequent and non-subsequent metastasis groups, respectively. The patient selection flow chart is shown in Figure 4. Table 1 shows the characteristics of these patients that were finally included, where the average tracer radioactivity at the time of administration in the PET examination was 261.4 MBq, the average blood glucose level was 104.8 mg/dL, and the average waiting time was 54.7 min. Among these features extracted from the primary tumor region on the PET images, 148 independent features showed a statistically significant difference (*p* < 0.05 in Welch’s *t*-test) between the subsequent and non-subsequent metastasis groups. Of these 148 features, 1 was a shape feature, 2 were first-order features, and the remaining 145 were texture features. Texture features obtained from all matrix types showed statistically significant differences, with GLCM providing the most features and GLRLM the least. The texture features extracted from the original images showed the most significant differences, while those obtained from the WL_LLL image showed the second highest number. All of the bins provided at least one texture feature that showed a statistically significant difference, with the texture features composed of large SUV differences in bin 10 also showing significant differences. Table 2 shows the types of these originally selected 148 features. LASSO regression was performed on 294 features, and 7 features were finally selected for creating the ML model. All seven features were texture features: three were GLSZM, three were NGTDM, and one was GLCM. Four of these features were extracted from the original image, two from the WL_LLL image, and one from the WL_HHH image. The bins ranged from 0.02 to 10. Table 3 shows the seven selected features and their coefficients. The prediction accuracy metrics for the various ML models applied to the training data were an AUC of 0.81–1.00, accuracy of 72.7–96.1%, sensitivity of 11.1–92.9%, specificity of 80.4–100.0%, PPV of 60.0–100.0%, and NPV of 77.2–96.0%. The prediction accuracies on the test data were an AUC of 0.81–1.00, accuracy of 72.7–96.1%, sensitivity of 11.1–92.9%, specificity of 80.4–100.0%, PPV of 60.0–100.0%, and NPV of 77.2–96.0% for the various ML models. The AUCs for the test data were considered good or excellent, with particularly high values of 0.977 for the random forest model and 0.918 for the support vector machine model. Accuracy and sensitivity also showed high values, but sensitivity was low. The results for these training and test data are shown in Table 4, and the ROC curves of the various ML models are shown in Figure 5 and Figure 6. Evaluation of the ML model performance without applying LASSO regression revealed suboptimal metrics on the test dataset, with AUC ranging from 0.485 to 0.721, accuracy from 53.75% to 75.63%, sensitivity from 23.06% to 63.06%, specificity from 55.63% to 94.89%, PPV from 36.08% to 62.67%, and NPV from 69.71% to 83.47% (see Table 5). These results were inferior to those obtained with LASSO-regularized models, underscoring the necessity of incorporating LASSO regression for improved predictive performance.

## 4. Discussion

In this study, 148 radiomics features that could be useful for predicting subsequent metastasis showed statistically significant differences between the subsequent and non-subsequent metastasis groups. Most of these features were texture features that quantitatively evaluated the metabolic heterogeneity of the region of interest in the PET images, suggesting that heterogeneity within the tumor is a factor influencing the subsequent metastasis of early OSCC. Many researchers have reported that metabolic heterogeneity within a tumor is associated with tumor malignancy, including the possibility of lymph node metastasis [23,25,26,27], which is consistent with our results. The features “bins 0.01 original Histogram Median” and “bins 0.01 WL_HHH Histogram Skewness”, both of which are first-order features, were confirmed to be predictors of subsequent metastasis. The feature “bins 0.01 original Histogram Median” was significantly higher in the subsequent metastasis group than in the non-metastasis group. In addition, 17 of the texture features focused on the intensity of the SUV, with these including “GLDM Small Dependence High Gray Level Emphasis”, “GLSZM High Gray Level Zone Emphasis”, and “GLRLM Short Run High Gray Level Emphasis”. Among these features, 16 tended to be higher in SUV in the subsequent metastasis group. Many researchers have reported that high intratumoral metabolism is also a factor in determining tumor malignancy and lymph node metastasis [28,29], which is consistent with our results. Among the shape features, “surface volume ratio” was identified as a feature that predicts subsequent metastasis. This value was significantly lower in the subsequent metastasis group; that is, the surface was smaller in relation to the volume, suggesting that the tumor was not spread thinly on the surface but had a tendency to invade. The seven features selected for constructing the ML model all focus on the heterogeneity of the SUVs. Metabolic heterogeneity within a tumor may be caused by the coexistence of different tissues. In OSCC, the primary tumor and metastatic lymph nodes often have necrotic areas that do not undergo metabolism, and the coexistence of these areas with areas of live tumor tissue may result in metabolic heterogeneity within the tumor. However, this study focused on early-stage cancer, and it is unlikely that lesions contained many areas of necrosis. Histopathological studies have shown that perineural invasion, lymphovascular invasion, depth of invasion, and tumor budding are factors that cause metastasis [30]. Among these, tumor budding is in accordance with both the metabolic heterogeneity shown by the PET radiomics and the histopathological findings that cause subsequent metastasis.

In recent years, many researchers have reported that tumor budding affects lymph node metastasis [31,32,33,34]. Tumor budding indicates the presence of discrete tumor masses at the tumor margin, indicating tumor cells intermingled with normal tissue. If we consider this in respect to the metabolism shown by PET, it seems that tumor metabolism and normal tissue metabolism are intermingled, which may result in metabolic heterogeneity reflecting the degree of budding at the lesion margin. However, because the early OSCC lesions targeted in this study were small, it is possible that marginal elements were averaged along with the lesion interior because of the low spatial resolution of the PET, and that metabolic heterogeneity in the margin due to tumor budding was averaged over the entire tumor. There are several previous studies on the prediction of subsequent metastasis or occult lymph node metastasis of OSCC. The performance metrics of these studies are shown in Table 6. Konishi et al. used radiomics analysis of preoperative intraoral ultrasonography to predict subsequent metastasis [11]. They reported extremely high accuracy with AUCs of 0.990–0.995, an accuracy of 93.8–95.0%, a sensitivity of 75.0–95.0%, and a specificity of 95.0–100.0%. This suggests that it is possible to predict subsequent metastasis with very high accuracy if radiomics features are extracted from images with high spatial resolution such as ultrasonography images. A method for predicting occult metastasis in early tongue SCC using magnetic resonance imaging (MRI) radiomics analysis was reported by Yuan et al. [12]. They reported an AUC of 0.802, accuracy of 74.1%, sensitivity of 63.3%, and specificity of 82.1%. Kudoh et al. reported a study that evaluated the accuracy of cervical lymph node metastasis prediction using radiomics analysis of PET images of tongue SSC, although their study was not limited to early stages [13]. They reported an AUC of 0.79, accuracy of 68%, sensitivity of 65%, and specificity of 70%. Our results demonstrated diagnostic accuracy comparable to or exceeding that of previous studies. This improvement is likely attributable to the number and diversity of radiomic features extracted in our study. While prior studies utilized 86 features (Yuan et al. [12]), 786 features (Wang et al. [14]), and 476 features (Kudo et al. [18]), our approach incorporated a design that extracted features from multiple bins, resulting in a total of 2993 features. This enriched feature set enabled the selection of predictive features with reduced image noise, thereby contributing to enhanced diagnostic performance compared to earlier research. In addition, our selection of a large number of features to cover the texture pattern of the metabolic distribution within the tumor appears to have led to the creation of ML models with similar or higher diagnostic accuracy compared to these previous studies. However, the fact that this accuracy was lower than that of intraoral ultrasonography may be due to the inherent low spatial resolution or metabolic distribution of PET, which may be unsuitable for capturing histopathological features that cause subsequent metastasis. In the future, further elucidation of the mechanism of subsequent metastasis will be required through more detailed research using ultrasonography, PET, or other modalities, and new histopathological findings.

There are three limitations to this research. First, this study was conducted at a single institution, and the number of subjects was small at 109 patients. Despite the high prediction accuracy of the ML models created in this study, it is necessary to train and test them using multicenter data to achieve higher accuracy and robustness. Multicenter research requires better standards for PET testing protocols across facilities to improve accuracy. Second, the low spatial resolution of the PET images may not accurately reflect the metabolic distribution of the tumor. If future developments in PET equipment allow texture features of metabolic distributions to be obtained from images with higher spatial resolution, it may be possible to capture metabolic features that lead to subsequent metastasis and obtain higher diagnostic accuracy. Finally, the SUV threshold of 2.5 used for lesion segmentation may not be ideal for the purpose of understanding lesion metabolism. Mittlmeier et al. reported an SUV threshold optimized for tumor differentiation [35], and there may also be an optimal SUV threshold for predicting subsequent metastasis. In particular, the lesions investigated in this study were small, and a lower SUV threshold may have been necessary to capture the metabolic features at the lesion margin. Wang et al. reported a method for predicting metastatic lymph nodes using MRI radiomics analysis of tongue cancer tumor areas and their margins [14], reporting an AUC of 0.872, accuracy of 87.34%, sensitivity of 78.78%, and specificity of 93.47%. Evaluations that include the margins have improved accuracy in studies using other modalities, and setting a region of interest that includes the margins by lowering the SUV threshold may improve prediction accuracy. We would like to increase the number of cases and investigate these issues in the future.

## 5. Conclusions

This study confirmed that a preoperative ^18^F-FDG PET examination can be a useful tool for predicting subsequent metastasis of OSCC through radiomics analysis. In the future, we would like to clarify the radiomics features or their combinations that more clearly reflect the metabolic distribution characteristics of those early OSCC lesions that result in subsequent metastasis.

Since this study targets medical data, such as cancer datasets, even minor misclassifications can have critical consequences. Therefore, further efforts to enhance accuracy are required, including the incorporation of novel image processing techniques and feature extraction methods, as suggested in recent radiomics papers [14,36]. Further studies are warranted to explore and enhance the potential utility of radiomics-based machine learning approaches.

## Figures and Tables

**Table 1:** Characteristics of the lymph nodes included in the dataset used in this study.

	Late Cervical Lymph Node Metastasis		Total (n = 109)
	Absent (n = 78)	Present (n = 31)	
Average age	68.9	68.6	68.8
Sex			
Male	42	18	60
Female	36	13	49
Site of Primary tumor			
Tongue	44	18	62
Floor of oral mouth	5	2	7
Gingiva of maxilla	6	8	14
Gingiva of mandible	11	2	13
Buccal mucosa	11	1	12
Palate	0	0	0
Lip	1	0	1
pT classification			
pT1	42	16	58
pT2	36	15	51

**Table 2:** Numbers and types of radiomics features showing significant differences according to Welch’s *t*-test.

Feature	N = 148
Feature type	
Shape feature	1
First-order feature	2
Texture feature	145
Texture features by matrix	
GLCM	48
GLDM	37
GLRLM	11
GLSZM	33
NGTDM	16
Image	
Original image	61
Wavelet HHH image	37
Wavelet LLL image	49
Bins	
0.01–0.05	34
0.1–0.5	49
1–5	55
10	9

**Table 3:** The seven features selected by LASSO regression and their respective coefficients.

Feature	Coefficient
bins 0.03 original GLCM MCC	0.00592462
bins 1 original GLSZM Small Area Emphasis	0.00803585
bins 2 original NGTDM Strength	0.0149423
bins 10 WL_LLL NGTDM Strength	0.0172186
bins 0.02 WL_HHH GLSZM Size Zone Non Uniformity Normalized	0.0256454
bins 2 WL_LLL NGTDM Contrast	0.0558602
bins 1 original GLSZM Zone Percentage	0.0579938

**Table 4:** Performance of the machine learning models on the training and test cohorts.

Cohort	ML Model	AUC	Accuracy (%)	Sensitivity (%)	Specificity (%)	PPV (%)	NPV (%)
		(95% CI)	(95% CI)	(95% CI)	(95% CI)	(95% CI)	(95% CI)
Training cohort (n = 76)	LR	0.821 (0.735–0.906)	72.73 (62.78–82.67)	48.00 (28.42–67.58)	84.62 (74.81–94.42)	60.00 (38.53–81.47)	77.19 (66.30–88.09)
	SVM	0.928 (0.870–0.986)	88.31 (81.14–95.49)	59.09 (38.55–79.64)	100.00 (100.00–100.00)	100.00 (100.00–100.00)	85.94 (77.42–94.45)
	RF	0.997 (0.986–1.000)	96.10 (91.78–100.00)	92.86 (83.32–100.00)	97.96 (94.00–100.00)	96.30 (89.17–100.00)	96.00 (90.57–100.00
	NB	0.830 (0.747–0.914)	75.32 (65.70–84.95)	65.39 (47.10–83.67)	80.39 (69.50–91.29)	62.96 (44.75–81.18)	82.00 (71.35–92.65)
	KNN	0.813 (0.726–0.900)	90.91 (84.49–97.33)	11.11 (0.00–25.63)	98.31 (95.01–100.00)	66.67 (13.32–100.00)	78.38 (69.00–87.76)
Test cohort (n = 33)	LR	0.821 (0.688–0.954)	81.25 (67.73–94.77)	50.00 (9.99–90.01)	88.46 (76.18–100.00)	5 0.00 (9.99–90.01)	88.46 (76.18–100.00)
	SVM	0.918 (0.822–1.000)	84.38 (71.79–96.96)	55.566 (23.09–88.02)	95.65 (87.32–100.00)	83.33 (53.51–100.00)	4.62 (70.75–98.48)
	RF	0.977 (0.925–1.000)	87.50 (76.04–98.96)	100.00 (100.00–100.00)	86.21 (73.66–98.76)	42.86 (6.20–79.52)	100.00 (100.00–100.00)
	NB	0.815 (0.680–0.949)	78.13 (63.80–92.45)	100.00 (100.00–100.00)	74.07 (57.54–90.60)	41.67 (13.77–69.56)	100.00 (100.00–100.00)
	KNN	0.804 (0.666–0.941)	62.50 (45.73–79.27)	7.69 (0.00–22.18)	100.00 (100.00–100.00)	100.00 (100.00–100.00)	61.29 (44.14–78.44)

ML: machine learning; LR: logistic regression; SVM: support vector machine; RF: random forest; NB: naive Bayes; KNN: k-nearest neighbor; AUC: area under the curve; PPV: positive predictive value; NPV: negative predictive value.

**Table 5:** Performance of the machine learning models without feature selection using LASSO regression on the training and test cohorts.

Cohort	ML Model	AUC	Accuracy (%)	Sensitivity (%)	Specificity (%)	PPV (%)	NPV (%)
		(95% CI)	(95% CI)	(95% CI)	(95% CI)	(95% CI)	(95% CI)
Training cohort (n = 76)	LR	0.969 (0.956–0.982)	90.65 (86.97–94.33)	70.06 (60.70–79.43)	98.51 (96.59–100.00)	95.45 (89.81–100.00)	89.53 (85.82–93.23)
	SVM	0.726 (0.604–0.848)	69.35 (56.02–82.68)	67.30 (61.86–72.74)	69.74 (51.18–88.31)	49.99 (36.59–63.38)	83.60 (77.39–89.81)
	RF	0.985 (0.977–0.993)	94.29 (92.84–95.73)	84.51 (78.51–90.51)	97.86 (95.99–99.72)	93.80 (88.33–99.26)	94.52 (92.98–96.07)
	NB	0.835 (0.822–0.848)	75.39 (72.90–77.88)	74.97 (69.28–80.66)	75.65 (71.18–80.13)	55.54 (51.02–60.06)	88.17 (84.92–91.42)
	KNN	0.770 (0.733–0.806)	75.58 (72.09–79.08)	25.22 (21.51–28.93)	97.04 (93.53–100.00)	80.45 (61.42–99.48)	75.27 (72.52–78.02)
Test cohort (n = 33)	LR	0.721 (0.634–0.808)	68.13 (58.46–77.79)	50.39 (15.34–85.43)	77.25 (56.11–98.39)	46.15 (30.72–61.58)	81.56 (68.16–94.96)
	SVM	0.485 (0.277–0.692)	53.75 (41.67–65.83)	50.33 (24.47–76.20)	55.63 (37.72–73.54)	36.08 (16.11–56.04)	69.71 (56.51–82.91)
	RF	0.642 (0.585–0.698)	70.00 (59.88–80.12)	25.98 (17.33–54.63)	84.35 (76.56–92.14)	48.10 (24.41–71.78)	76.30 (66.21–86.39)
	NB	0.654 (0.567–0.742)	63.13 (55.66–70.59)	63.06 (46.62–79.49)	62.97 (54.01–71.93)	37.19 (28.25–46.14)	83.47 (77.55–89.38)
	KNN	0.694 (0.580–0.808)	75.63 (69.87–81.38)	23.06 (6.44–39.67)	94.89 (90.47–99.32)	62.67 (29.38–95.96)	77.16 (73.06–81.26)

ML: machine learning, LR: logistic regression, SVM: support vector machine, RF: random forest, NB: naive Bayes, KNN: k-nearest neighbor, AUC: area under the curve, PPV: positive predictive value, NPV; negative predictive value.

**Table 6:** Performance of machine learning models in previous studies.

Authors	Year	Modality	AUC	Accuracy (%)	Sensitivity (%)	Specificity (%)
Konishi et al. [11]	2023	Ultrasonography	0.967	95.0	90.0	96.7
Yuan et al. [12]	2021	MRI	0.802	74.1	63.3	82.1
Kudoh et al. [18]	2023	PET	0.790	68.0	65.0	70.0
Wang et al. [20]	2024	MRI	0.872	87.34	78.78	93.47

AUC: area under the curve.

## Data Availability

The data presented in this study are available on request from the corresponding author due to regulations in institutional research ethics review.

## Abbreviations

The following abbreviations are used in this manuscript:

OSCC	Oral squamous cell carcinoma
PET	Positron emission tomography
ML	Machine learning
GLCM	Gray level co-occurrence matrix
GLDM	Gray level dependence matrix
GLRLM	Gray level run length matrix
GLSZM	Gray level size zone matrix
NGTDM	Neighboring gray tone difference matrix
LR	Logistic regression
SVM	Support vector machine
RF	Random forest
NB	Naive Bayes
KNN	K-nearest neighbor
AUC	Linear dichroism
